# Advancing Medical Education: Performance of Generative Artificial Intelligence Models on Otolaryngology Board Preparation Questions With Image Analysis Insights

**DOI:** 10.7759/cureus.64204

**Published:** 2024-07-09

**Authors:** Emma Terwilliger, George Bcharah, Hend Bcharah, Estefana Bcharah, Clare Richardson, Patrick Scheffler

**Affiliations:** 1 Otolaryngology, Mayo Clinic Alix School of Medicine, Scottsdale, USA; 2 Otolaryngology, Andrew Taylor Still University School of Osteopathic Medicine, Mesa, USA; 3 Otolaryngology, Arizona State University, Tempe, USA; 4 Otolaryngology, Phoenix Children's Hospital, Phoenix, USA

**Keywords:** education, artificial intelligence, board exam, otolaryngology, chatgpt

## Abstract

Objective

To evaluate and compare the performance of Chat Generative Pre-Trained Transformer (ChatGPT), GPT-4, and Google Bard on United States otolaryngology board-style questions to scale their ability to act as an adjunctive study tool and resource for students and doctors.

Methods

A 1077 text question and 60 image-based questions from the otolaryngology board exam preparation tool BoardVitals were inputted into ChatGPT, GPT-4, and Google Bard. The questions were scaled true or false, depending on whether the artificial intelligence (AI) modality provided the correct response. Data analysis was performed in R Studio.

Results

GPT-4 scored the highest at 78.7% compared to ChatGPT and Bard at 55.3% and 61.7% (p<0.001), respectively. In terms of question difficulty, all three AI models performed best on easy questions (ChatGPT: 69.7%, GPT-4: 92.5%, and Bard: 76.4%) and worst on hard questions (ChatGPT: 42.3%, GPT-4: 61.3%, and Bard: 45.6%). Across all difficulty levels, GPT-4 did better than Bard and ChatGPT (p<0.0001). GPT-4 outperformed ChatGPT and Bard in all subspecialty sections, with significantly higher scores (p<0.05) on all sections except allergy (p>0.05). On image-based questions, GPT-4 performed better than Bard (56.7% vs 46.4%, p=0.368) and had better overall image interpretation capabilities.

Conclusion

This study showed that the GPT-4 model performed better than both ChatGPT and Bard on the United States otolaryngology board practice questions. Although the GPT-4 results were promising, AI should still be used with caution when being implemented in medical education or patient care settings.

## Introduction

Artificial intelligence (AI) is a multimodal computer-based program that is designed to “think” like a human [1]. AI programs have been constructed with multiple techniques to derive well-automated answers, and most commonly include machine learning, neural networks and deep learning, data mining/knowledge discovery and advanced analytics, rule-based modeling and decision-making, fuzzy logic-based approach, knowledge representation/uncertainty reasoning and expert system modeling, case-based reasoning, text mining and natural language processing, visual analytics/computer vision and pattern recognition, and hybridization/searching/and optimization [2]. These various constructs allow AI to handle real-world complex problems that require several dimensions of thought. 

The first AI program was developed in 1956 as a Dartmouth summer research project [3]. This project proposed that humanistic ways of thinking could be replicated and simulated by algorithms. Since that first description, the field has rapidly evolved and expanded. AI is now widely accessible, and with the advent of universally accessible programs, it is becoming more commonplace. One of the widely recognized AI tools, Chat Generative Pre-Trained Transformer (ChatGPT), has gained significant popularity, reaching over one million users in the first five days of its launch [4]. ChatGPT utilized 175 billion parameters for language processing, allowing it to answer a wide range of user prompts [1,5]. These prompts range from checking grammatical errors to creating a differential diagnosis based on listed symptoms. Recently, ChatGPT came out with a newer, updated, and more powerful language model called GPT-4. In testing, it has significantly outperformed ChatGPT on multiple standardized examinations, including the American Bar Exam (90th vs 10th percentile) and the Verbal Graduate Record Examination (GRE) (99th percentile vs 63rd percentile) [6]. In addition, GPT-4 now has the ability to handle uploaded documents, produce original documents, create graphs, read images, and browse the web, which is not available in ChatGPT [7].

Since the release of ChatGPT and its growing popularity, other competitive AI models have been released in response. On March 21st, 2023, Google released its first competitor called Google Bard [8]. Although the first users had joined a waiting list, it is now publicly accessible for use. Google Bard uses real-time information from Google searches when generating responses, unlike ChatGPT that answers questions based on data prior to 2021 [9]. Although its launch was successful with over 142 million visits in May, there are still many updates being implemented. In all, Google Bard is still considered “experimental” and many new forms of this application are being built [10]. 

In otolaryngology, physicians are already using AI models in clinical practice to aid in recognizing auditory brainstem response waveforms, identifying malignant tissue on pathology slides, and predicting the prognosis of patients with varying diseases. A promising example of its use is the almost 100% accuracy of diagnosing vocal cord pathologies when AI programs are used in conjunction with voice analysis and videostroboscopy images [11]. Surveys have also shown that the majority of otorhinolaryngologists (78%) agreed that AI could potentially have useful clinical indications [12].

The advent of AI has naturally raised questions about how generative AI models can be used as tools in the medical field, such as for study purposes. Board examinations represent the key milestones of medical knowledge for most medical students and residents. The ability to answer board-style questions is often multidimensional, requiring trainees to use both knowledge and judgment to come to a conclusive answer. BoardVitals offers 1248 practice questions that simulate the question style and content of the otolaryngology board exam taken at the end of the residency. The questions are filtered in three ways: question status, difficulty level, and subject [13]. This study aimed to investigate the accuracy, and thus the utility of the three predominant AI models (ChatGPT, GPT-4, or Google Bard) for querying boards-style, otolaryngology-specific medical knowledge. In addition, the newly released image-interpretation capabilities of GPT-4 and Bard, issued on 09/25/2023 and 09/19/2023, respectively, were tested using image-based questions from the practice set [7,14]. 

## Materials and methods

This study was determined to be exempt from Institutional Board Review. ChatGPT, GPT-4, and Google Bard were individually queried using question sets from the common otolaryngology board exam preparation tool BoardVitalsTM (boardvitals.com) [13]. A BoardVitalsTM representative approved the use of questions for research purposes as long as the following parameters were followed: a membership was individually purchased, no questions were saved or displayed in the study, and appropriate measures were taken to protect the integrity of the question database (which were all adhered to). The basic ChatGPT version and Google Bard were free, whereas GPT-4 membership was purchased at $20/month [1]. A monthly membership to BoardVitals was also purchased, at $209. Other options for purchasing this study aid are six months/$549 or three months/$349. This program is often covered by many training programs for residents to use. Mayo Clinic IRB Wizard was used to determine that no IRB was required for this study.

Boards-style question generation  

Quizzes were created on BoardVitals consisting of unanswered questions about one subject matter at one specific level of difficulty. Examination sections included allergy, endocrine, head and neck, laryngology, otology, pediatrics, pharmacology, plastics, rhinology, and sleep and difficulty levels included easy, moderate, and hard. For example, one quiz set consisted of 19 unanswered allergy question prompts with a difficulty level of Hard.  

Questions were excluded if at least one of the AI models responded with an error message (for example: “content violation” or “not enough information”). Image questions were excluded when comparing overall performance since not all the tested AI models have image-reading capabilities (i.e., ChatGPT). Image-based questions were analyzed separately and compared between Bard and GPT-4. 

Input into artificial intelligence programming  

Each question was entered into each of the three studied AI platforms as an individual prompt. Each prompt was started by asking the chatbot the following question: “Please select the correct answer and provide an explanation.” The questions were then manually typed into the chat with an identical format to the BoardVitals question. The question was then answered on BoardVitals using the choice made by ChatGPT, GPT-4, or Google Bard. If the answer choice was incorrect, the output was recorded as false. True was recorded if the answer was correct. Initially, all questions with images or questions that could not be completed by simple prompting into one or more of the platforms were excluded from the analysis.  

Following initial analysis, 60 randomly selected image-based questions (20 easy, 20 moderate, and 20 hard) were evaluated separately in GPT-4 and Google Bard, since both have an image input capability while ChatGPT does not. The text component of the question was provided to the chatbot, and the image related to the question was included as an attachment. The following prompt was used: “Choose the best answer. Make sure to describe and use the image provided in the question.” Answer choices were then inputted into BoardVitals and recorded as described above, and the AI models' explanation of the images was stored. All images used were deleted after the conclusion of the study.

Statistical analysis  

Statistical analysis and data visualization were performed in R Studio. Chi-squared analysis was done to compare the performance of the three individual AI models, with p<0.05 indicating statistical significance. 

## Results

Out of 1248 possible questions, 1077 were included and 171 were excluded. Reasons for exclusion included image-based questions (n=159) as well as a response of “not enough information,” “violated content policy,” or “could not answer” (n=12). 

Overall, ChatGPT scored 595/1077 (55.3%), GPT-4 scored 847/1077 (78.7%), and Bard scored 664/1077 (61.7%). Bard’s overall score was significantly higher than ChatGPT’s (p<0.01), but GPT-4 performed better than both ChatGPT (p<0.001) and Bard (p<0.001). 

**Figure 1:**
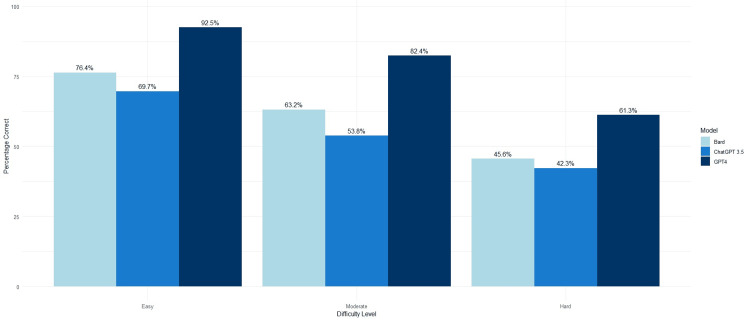
Performance of ChatGPT-3.5, GPT-4, and Google Bard by question difficulty (n=1077) ChatGPT: Chat Generative Pre-Trained Transformer

When stratifying questions by difficulty level, all three AI models performed best on easy questions (ChatGPT: 69.7%, GPT-4: 92.5%, and Bard: 76.4%) and worst on hard questions (ChatGPT: 42.3%, GPT-4: 61.2%, and Bard: 45.6%). Across all three difficulty levels, GPT-4 performed best, resulting in higher overall correct scores than Bard and ChatGPT (p<0.0001 for both). The performances of each AI model by question difficulty are displayed in Figure 1 and compared in Table 1. 

**Table 1:** Comparison of AI model performance by question difficulty ^*^indicates significance AI: artificial intelligence

Difficulty	Questions	ChatGPT-3.5	GPT-4	Bard	P-values		
					ChatGPT-3.5 vs GPT-4	ChatGPT-3.5 vs Bard	GPT-4 vs Bard
Easy	360	251 (69.7%)	333 (92.5%)	275 (76.4%)	<0.001*	0.053	<0.001*
Moderate	353	190 (53.8%)	291 (82.4%)	223 (63.2%)	<0.001*	<0.05*	<0.001*
Hard	364	154 (42.3%)	223 (61.3%)	166 (45.6%)	<0.001*	0.411	<0.001*
Overall	1077	595 (55.3%)	847 (78.7%)	664 (61.7%)	<0.001*	<0.01*	<0.001*

**Figure 2:**
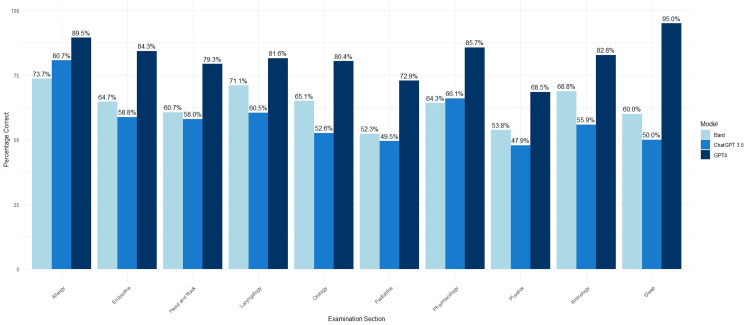
Performance of ChatGPT-3.5, GPT-4, and Google Bard by question examination section (n=1077) ChatGPT: Chat Generative Pre-Trained Transformer

When analyzing results by subspecialty section, ChatGPT and Bard performed best on the allergy section (80.7% vs 73.7%, p=0.503), while GPT-4 performed best on sleep (95%, p<0.001 compared to ChatGPT and Bard) followed by pharmacology (85.7%, p<0.05). Overall, GPT-4 outperformed ChatGPT and Bard in all subspecialty sections. Although it still performed better in the allergy section, the difference was not statistically significant (89.5% vs 80.7% and 73.6%, p≥0.05 for all). ChatGPT and GPT-4 performed worst on plastics (47.9% vs 68.5%, p<0.001), and Bard performed worst on pediatrics (52.3%, p<0.01 compared to GPT-4). The performances of all AI models by subspecialty section are displayed in Figure 2 and compared in Table 2. 

**Table 2:** Comparison of AI model performance by subspecialty section ^*^indicates significance ChatGPT: Chat Generative Pre-Trained Transformer

Section	Questions	ChatGPT-3.5	GPT-4	Bard	P-values		
					ChatGPT-3.5 vs GPT-4	ChatGPT-3.5 vs Bard	GPT-4 vs Bard
Allergy	57	46 (80.7%)	51 (89.5%)	42 (73.7%)	0.293	0.503	0.053
Endocrine	51	30 (58.8%)	43 (84.3%)	33 (64.7%)	<0.01*	0.684	<0.05*
Head & neck	150	87 (58.0%)	119 (79.3%)	91 (60.7%)	<0.001*	0.724	<0.001*
Laryngology	76	46 (60.5%)	62 (81.6%)	54 (71.1%)	<0.01*	0.231	0.182
Otology	209	110 (52.6%)	168 (80.4%)	136 (65.1%)	<0.001*	<0.01*	<0.001*
Pediatrics	107	53 (49.5%)	78 (72.9%)	56 (52.3%)	<0.001*	0.785	<0.01*
Pharmacology	56	37 (66.1%)	48 (85.7%)	36 (64.3%)	<0.05*	1	<0.05*
Plastics	238	114 (47.9%)	163 (68.5%)	128 (53.8%)	<0.001*	0.233	<0.01*
Rhinology	93	52 (55.9%)	77 (82.8%)	64 (68.8%)	<0.001*	0.096	<0.05*
Sleep	40	20 (50.0%)	38 (95.0%)	24 (60.0%)	<0.001*	0.500	<0.001*

From the included questions containing images, GPT-4 scored 34/60 (56.7%). GPT-4 overall was capable of interpreting a wide range of images, from radiologic images and histologic slides to schematics and physical exam images. Conversely, Bard faced considerable challenges with interpreting images, returning error messages (“I can’t process this file”) on 32/60 of the questions despite being prompted more than once. With the remaining 28 questions, it obtained a score of 13/28 (46.4%, p=0.368 compared to the GPT-4 score). Sample responses from GPT-4 and Bard can be found in Table 3. 

**Table 3:** Sample responses from GPT-4 and Bard on image-based questions

AI model	Response to image-based question
GPT-4	"The image displays a computed tomography (CT) scan of the head, focusing on the region of the parotid glands. The parotid glands on both sides seem to be enlarged with well-defined boundaries.”
	“The image provided is a computed tomography (CT) scan that appears to highlight a section of the ear. The red arrow points to a structure that seems to be the facial nerve canal.”
	“The image provided is a histological representation of thyroid tissue. The appearance of the cells and the arrangement of the follicles is suggestive of papillary thyroid carcinoma, which is the most common type of thyroid cancer. The nuclear features such as overlapping nuclei, ground-glass appearance, nuclear grooves, and occasional intranuclear inclusions support this diagnosis. Papillary thyroid carcinoma is associated with radiation exposure, including that from the Chernobyl nuclear disaster.”
	“The image shows a drain with milky fluid suggestive of chyle, and given the history and the elevated triglyceride content, this is consistent with a chyle leak.”
Bard	“Congo red is a special stain that stains amyloid deposits. Amyloid deposits are typically seen in medullary thyroid carcinoma (MTC).”
	“The dark, “motor oil” fluid is a characteristic feature of adenoid cystic carcinoma. The H&E stain shows a biphasic tumor with epithelial and myoepithelial elements. The epithelial component forms a cribriform pattern, which is another characteristic feature of adenoid cystic carcinoma.”
	“The audiogram in the image shows a conductive hearing loss, which is consistent with otosclerosis. The absent stapedial reflex also supports the diagnosis of otosclerosis.”
	“Adenoid cystic carcinoma is a malignant salivary gland tumor that is characterized by a biphasic appearance, with epithelial and myoepithelial elements. The epithelial component forms a cribriform pattern, which is seen in the image. The tumor cells are also small and uniform, which is another characteristic feature of adenoid cystic carcinoma.”

## Discussion

The use of AI is actively being evaluated across different levels and fields of medicine. In recent studies, AI tools achieved above the median score for the Medical College Admission Test (MCAT) and passing scores on the United States Medical Licensing Exam (USMLE) step 1 exam along with medical board exams, including cardiology and neurosurgery [15-18]. This study represents the first description of the performance of these three AI modalities on over 1000 United States board-style otolaryngology examination questions. Two prior studies have evaluated ChatGPT and GPT-4 performance on the otolaryngology board exams in Germany and Canada, respectively [19,20]. Among these studies, ChatGPT performed worse (57%) than GPT-4 (average of 72%), which was consistent with the findings of this study (55.3% vs 78.7%). 

Notably, GPT-4 (78.7%) performed better than Bard (61.7%, p<0.001) and ChatGPT-3.5 (55.3%, p<0.001) across all difficulty levels, on all examination sections, and with questions incorporating images. Compared to other AI models, GPT-4 has undergone more extensive dataset training and features improved algorithms and higher parameters that deliver more accurate outputs, and many studies outline its performance on medical examinations [21]. In other studies, GPT-4 significantly outperformed ChatGPT and Bard on a neurosurgery oral boards question bank with a score of 82.6% compared to 62.4% and 44.2%, respectively [17]. GPT-4 also performed up to 24.1% and 32.1% better when compared to ChatGPT in a set of Polish and Japanese medical examination questions, respectively [22,23]. GPT-4 surpassed the average score of fifth-year orthopedic surgery trainees on the orthopedic in-training examination, with ChatGPT performing above the first-year trainee average [24]. GPT-4’s ability to achieve consistently high performance across medical disciplines, including in otolaryngology, supports its potential in the medical education field. Through targeted training tailored to different medical specialties and close validation by medical experts, GPT-4 models have the potential to transform into reliable, evidence-based tools that can be used in medical training, patient education, diagnosis guidance, and outcome prediction. 

When analyzing the AI tools that scored lower than GPT-4 (i.e., ChatGPT and Bard), it is relevant to note that while these tools are not explicitly designed for medical testing or education, they still achieved a >50% score on otolaryngology board-style questions. ChatGPT and Bard achieved their highest performance on allergy questions (80.7% and 73.7%, p=0.503), which is a consistent finding in other studies [19]. This section involves a larger proportion of questions with definite answers, such as immunologic and cellular pathways, compared to other sections that present various patient findings with multiple probable answers. These AI models performed poorly on more complex sections, likely due to the requirement for more nuanced decision-making and interdisciplinary clinical judgment. This distinction highlights the difference between simple data retrieval, where many AI models excel, and more intricate second-level reasoning, which is still being refined in the AI realm. The variability in performance across the different sections shows that models like ChatGPT and Bard might be more suited for educational material that requires less clinical judgment and multi-dimensional thinking. With this in mind, Google has already released that they are working with medical professionals and researchers to develop a new model called Med-PaLM 2 [25]. The goal of this model is to use expert-analyzed research to answer medical questions, draft responses, and summarize diseases. 

In comparing these models in more depth, Bard achieved higher scores compared to ChatGPT, performing better overall (61.7% vs 55.3%, p<0.01) and in the otology section (65.1% vs 52.6%, p<0.01). This was unexpected considering that ChatGPT significantly outperformed Bard across other specialty exams, like neurosurgery (62.4% and 44.2%, p=0.01) and radiology (87.11% vs 70.44%, p<0.0001) [17,26]. This may be a result of Bard’s recent update on 09/19/2023, following the publication of other studies. The update introduced the "most capable version of Bard" to date, featuring higher accuracy achieved after implementing user feedback into the model [14]. In addition, Bard, unlike ChatGPT, has direct access to the internet and uses its web searching tool as a basis for its response, giving it the advantage of getting the most current information. This capability has likely also improved as part of Bard’s feedback implementation system, or reinforcement learning on human feedback (RLHF), as Bard is becoming more focused on knowledge exploration and collaboration [14]. 

The newly released image processing tools within some AI models also create many new possibilities for multidimensional data inputs. It should be noted, however, that these tools are still in their early stages and require further feedback and training to be reliable. This is evidenced when comparing Bard and GPT-4, with GPT-4 scoring 56.7% on the set of 60 image questions and Bard 46.4% on the images it could interpret (p=0.368). Bard was unable to process >50% of image-based questions due to file processing errors. Although, for the images Bard processed, it had the capability of reading graphs, identifying histopathological findings, and interpreting models. Bard was unable to process any radiological images and often relied on the question stem over the image when answering the question. GPT-4 was able to identify histological patterns, but it showed an increase in capability by correctly delineating glands and sinuses on CT scans and interpreting images with physical exam findings (Table 3). Another recent study examined the image interpretation of GPT-4 on 46 Japanese otolaryngology exam questions; however, the error output was significant (89.6%), and the questions were translated to English that improved the error rate (5.8%) and accuracy (41.3%), pointing to the language limitations of such models [27]. The image processing capabilities of GPT-4 are slowly being used in clinical practice, where, for instance, GPT-4 was able to diagnose different forms of middle ear diseases at an overall accuracy of 82.1% using real patient otoscopic images [28]. While it did not perform at the level of otolaryngologists, the results point to the future of AI image interpretation in augmenting diagnosis accuracy, although more training and validation of such models is needed to reach that stage.

While the results of this study suggest AI as an emerging study adjunct, they also suggest caution regarding the use of AI (and especially more primitive AI models, like ChatGPT and Bard) as a primary study tool for medical students and residents. In real-world applications, achieving 50%-60% on an assessment does not correlate with competency on the topic. As a study outlet, AI may skew complex medical problems into more abstract and one-dimensional answers based solely on facts. While the explanation of AI may provide some factual insight into the question at hand, it may also provide false assumptions and biased responses based on the availability of data. It is also important to warn against the phenomenon of AI “hallucinations,” where language models can present erroneous information in a sensible or factual manner [29]. These hallucinations can be challenging to detect, especially for non-medical users or inexperienced medical trainees, and can provide inaccurate medical information that could compromise the educational experience and even patient care. Ultimately, these models may be useful as a primitive search engine for medical problems with definitive answers but have not yet provided consistent, reliable answers for complex medical problems that may be on medical board exams. 

This study has several limitations. First, as otolaryngology board questions are not open source, the data relies instead on board-style questions. While these were taken from one of the most popular commercially available board-study tools, they may not fully represent questions on the board exam themselves. In addition, there was limited data on the web about exact passing scores and score distributions on the otolaryngology board exam, making it challenging to compare the AIs’ performances to others taking the exam. An additional limitation was the inability to properly compare the image-based questions across the AI models since ChatGPT did not have that capability and Bard had a considerable number of errors. Despite these factors, this study still represents the first investigation into how multiple AI models perform on otolaryngology-specific questions in the United States and represents valuable pilot data that may be expanded upon in the future. 

## Conclusions

This study shows that while current AI models demonstrate promise for use as study adjuncts in otolaryngology, they have not yet provided consistent, reliable answers for complex medical problems that may be on medical board exams. The most accurate AI modality based on these data was GPT-4, which performed best among different otolaryngology subspecialty topics, question difficulty levels, and questions including images compared to ChatGPT and Bard. Further research and expert validation of these AI tools may motivate their use in medicine or prompt the development of more medically oriented AI models.
